# Mid-Term Functional Recovery after Surgical Correction of ALCAPA: A 16-Year Single-Center Experience

**DOI:** 10.5761/atcs.oa.25-00181

**Published:** 2026-01-16

**Authors:** Mehmet B. Beyter, Eser Dogan, Osman N. Tuncer, Firat Ergin, Gulcin Kayan-Kasikci, Zulal Ulger, Erturk Levent, Yuksel Atay

**Affiliations:** 1 Department of Pediatrics, Division of Pediatric Cardiology, Ege University School of Medicine, Izmir, Turkey; 2 Department of Cardiovascular Surgery, Ege University School of Medicine, Izmir, Turkey

**Keywords:** anomalous origin of the left coronary artery from the pulmonary artery, left ventricular function, mitral regurgitation

## Abstract

**Purpose:**

This study aimed to assess perioperative features, early postoperative outcomes, and mid-term cardiac function in children with anomalous origin of the left coronary artery from the pulmonary artery (ALCAPA) undergoing surgical repair.

**Methods:**

A retrospective review of 23 patients treated surgically between 2007 and 2023 was conducted. Patients were categorized into infants (<1 year) and older patients (>1 year). Clinical, operative, and echocardiographic data were analyzed, including left ventricular ejection fraction (LVEF) and mitral regurgitation (MR). Follow-up evaluations were performed at 1 and 6 months postoperatively.

**Results:**

The median age at surgery was 9 months. Early mortality occurred in 17.4%, with no late deaths during follow-up. Preoperative LVEF was significantly lower in infants than in older patients (p = 0.013). Among 19 survivors, LVEF improved markedly by 1 month and normalized in all patients by 6 months. MR was present in 89.5% preoperatively, with 47.3% showing moderate to severe grades. At 6 months, MR improved in most cases, with only 2 patients exhibiting residual moderate regurgitation and no severe cases.

**Conclusions:**

ALCAPA is a rare but surgically correctable condition. Early surgical intervention leads to significant recovery of ventricular function and regression of MR within the first 6 postoperative months.

## Introduction

Anomalous origin of the left coronary artery from the pulmonary artery (ALCAPA) represents a rare congenital coronary anomaly, with an estimated incidence of 1 in 300000 live births, comprising 0.25%–0.5% of all congenital cardiac anomalies.^1,2)^ Originally described by pathologists in 1885, the first clinical case of the anomaly was reported in 1933 in a 3-month-old infant.^3,4)^ Due to this early report, the anomaly is also known as Bland–White–Garland syndrome.

ALCAPA may result in significant heart failure, life-threatening arrhythmias, and sudden cardiac death due to myocardial ischemia. Based on the variability in symptom onset and clinical findings, this anomaly is classified into 2 major forms: the infant type and the adult type. The infant type is characterized predominantly by early-onset signs of heart failure. In contrast, the adult type may present with a wide spectrum of manifestations ranging from asymptomatic cases to myocardial ischemia, syncope, and arrhythmias.^5)^

ALCAPA is associated with high mortality when not surgically treated during infancy, with up to 90% of affected infants dying within the first year of life if left untreated. These patients can rarely remain asymptomatic into adulthood; however, even in this subset, subclinical myocardial ischemia persists, and the incidence of sudden cardiac death is estimated to be 80%–90% at a mean age of 35 years.^6)^ Patients with reduced left ventricular function and more severe mitral regurgitation (MR) have been shown to experience higher perioperative and long-term mortality rates. These factors are important predictors of adverse outcomes and are critical considerations in the management and timing of surgical intervention for ALCAPA patients.^7)^

Given the high mortality and risk of severe cardiac complications associated with untreated ALCAPA, timely surgical intervention is essential. Various surgical techniques have been developed to restore a 2-coronary-artery system in affected patients, including coronary reimplantation, the Takeuchi procedure, and coronary artery bypass grafting (CABG).^8,9)^ This study aimed to evaluate the perioperative characteristics and postoperative outcomes of patients who underwent surgical treatment for this condition at our center.

## Materials and Methods

In this retrospective study, data were obtained from electronic health records and institutional clinical data repositories. We included all patients diagnosed with ALCAPA who underwent surgical treatment at our institution between 2007 and 2023. Clinical, procedural, and follow-up data were collected and comprehensively analyzed. The study was approved by the Ege University Medical Research Ethics Committee.

### Definitions

Routine 2-dimensional echocardiography and Doppler flow imaging were performed using ultrasound equipment (G.E. Vivid 7 and Vivid E9; GE Healthcare, Chicago, IL, USA) by a pediatric cardiologist. In selected cases, computed tomography or cardiac catheterization was performed to confirm the diagnosis. Left ventricular ejection fraction (LVEF) was used to assess ventricular function and was measured using the M-mode method. Patients with an LVEF of 55% or higher were considered to have normal ventricular function. Early mortality was defined as death occurring before hospital discharge or within 30 days post-surgery, whereas late mortality was defined as death occurring after discharge. Postoperative left ventricular function was considered normalized when LVEF was ≥55%. MR severity was classified into 4 grades—none, mild, moderate, and severe—based on the ratio of the regurgitant jet area to the left atrial area. Patients were stratified into 2 age groups for analysis: infants (aged less than 1 year) and children older than 1 year.

### Surgical techniques

All patients underwent median sternotomy under intratracheal general anesthesia. Cardiopulmonary bypass and systemic hypothermia were established. Following aortic cross-clamping, diastolic arrest was achieved using topical hypothermia and cardioplegia. In patients who underwent coronary reimplantation surgery, the main pulmonary artery was incised, and the left coronary artery was mobilized. It was subsequently reimplanted into the ascending aortic root through a fenestration. For the Takeuchi procedure, the main pulmonary artery was vertically incised, and a tunnel was created using an autologous pericardial patch to redirect the ostium of the left main coronary artery to the aorta. For CABG, the left internal mammary artery (LIMA) was used as a graft. The left coronary ostium was closed with an autologous pericardial patch. A distal anastomosis between the LIMA and the left anterior descending artery was performed. In all patients, the pulmonary artery incision was repaired using either polypropylene sutures or an autologous pericardial patch. Cardiopulmonary bypass and aortic cross-clamp times were measured and recorded for all patients. Mitral valvuloplasty was not performed in any case. In patients diagnosed with additional congenital cardiac defects, the corresponding repairs were performed concurrently. The choice of surgical technique was determined based on patient age, the position of the left coronary artery, and the surgical team’s experience.

### Follow-up

Postoperative requirements for mechanical ventilation and inotropic support, duration of cardiac intensive care unit (CICU) stay, total hospital stay, and early postoperative complications were evaluated. Follow-up echocardiographic examinations were scheduled at 1, 3, and 6 months after surgery, and annually thereafter. Echocardiography reports from outpatient follow-up visits of discharged patients were reviewed to assess ventricular function, mitral valve function, and any potential complications at 1 and 6 months postoperatively.

### Statistically analysis

Statistical analyses were performed using IBM SPSS Statistics for Windows, version 25.0 (IBM Corp., Armonk, NY, USA). Continuous variables were presented as medians with interquartile ranges (IQRs) due to non-normal distribution. Categorical variables were expressed as counts and percentages (n, %). Comparisons between infant (<1 year) and older (>1 year) patient groups were conducted using the Mann–Whitney U test for continuous variables and the chi-squared or Fisher’s exact test for categorical variables, as appropriate. Changes in LVEF across preoperative, 1-month, and 6-month time points were assessed using the Wilcoxon signed-rank test. Results were considered statistically significant when p <0.05.

## Results

A total of 23 patients diagnosed with ALCAPA who underwent surgical treatment at our center were included in the study. The median age at the time of surgery was 9 months (range: 1–411 months), and the surgical weight was 6.9 kg (range: 4.2–49 kg). The infant group consisted of 12 patients (52.1%), while the group older than 1 year included 11 patients, of whom 2 were adults (18 and 34 years old). Demographic data, presenting symptoms, and concomitant congenital heart diseases of the patients are presented in **Table 1**.

**Table 1:** Clinical and surgical data between infant and older patient groups

Variable	Total (n = 23) (100%)	Infant (n = 12) (52.2%)	Older (n = 11) (47.8%)
Gender (female)	14 (60.9%)	7 (58.3%)	7 (63.6%)
Surgical age (month)	9 (IQR: 88)	4 (IQR: 2.5)	92 (IQR: 133)
Surgical weight (kg)	6.9 (IQR: 16.5)	5.65 (IQR: 1.88)	22 (IQR: 31)
Symptoms			
Cardiac murmur	6 (26.1%)	2	4
Respiratory distress	6 (26.1%)	5	1
Feeding intolerance	4 (17.4%)	4	—
Growth failure	4 (17.4%)	1	3
Fatigue on exertion	2 (8.7%)	—	2
Sudden cardiac arrest	1 (4.3%)	—	1
Concomitant heart defects			
Patent ductus arteriosus	3 (13%)	3	—
Atrial septal defect	3 (13%)	3	—
Preoperative LVEF (%)	45 (IQR: 31)	35 (IQR: 27.50)^*^	56 (IQR: 30)
CPB time (min)	98 (IQR: 23)	107 (IQR: 26.75)	95 (IQR: 28)
ACC time (min)	84 (IQR: 22)	85 (IQR: 21)	79 (IQR: 25)
Ventilation duration (h)	45 (IQR: 150)	141 (IQR: 171.5)^*^	10 (IQR: 14)
Inotrope duration (h)	45 (IQR: 274)	138 (IQR: 258.75)^*^	4 (IQR: 17)
CICU stay (days)	4 (IQR: 14)	15.5 (IQR: 15)^*^	3 (IQR: 2)
Hospital stay (days)	12 (IQR: 16)	23.5 (IQR: 20.75)	8 (IQR: 5)

^*^p <0.05, statistically significant difference between infant and older groups.

LVEF: left ventricular ejection fraction; CPB: cardiopulmonary bypass; ACC: aortic cross-clamp; CICU: cardiac intensive care unit; IQR: interquartile range

Surgical interventions among the 23 patients included in the study comprised the Takeuchi procedure in 19 patients (82.6%), CABG in 3 patients, and coronary reimplantation in 1 patient. Preoperatively, 21 patients (91.3%) were hemodynamically stable and did not require respiratory support or inotropic therapy. The remaining 2 patients, both from the infant group, underwent surgery while intubated and receiving mechanical ventilation and inotropic support due to decompensated heart failure. Preoperative evaluation of ventricular function revealed reduced ventricular ejection fraction in 14 patients (60.9%). In infants, 11 out of 12 patients (91.7%) exhibited reduced ventricular function. There was a statistically significant difference in preoperative LVEF between infants and older patients (p = 0.013) (**Table 1**).

There were no significant differences between the 2 groups in terms of cardiopulmonary bypass time and aortic cross-clamp time. Postoperative data comparison revealed that the infant group had longer durations of mechanical ventilation, inotropic support, and CICU stay. However, there was no significant difference in overall hospital stay between the groups (**Table 1**).

Early postoperative mortality was observed in 4 patients, accounting for 17.4% of the study population. No mortality was recorded during the late postoperative follow-up period. Nineteen patients were discharged without complications and continued with outpatient follow-up. Detailed clinical characteristics and outcomes of the patients who experienced early postoperative mortality are presented in **Table 2**.

**Table 2:** Clinical characteristics and outcomes of patients with early postoperative mortality

Patient no.	Age (months)	Preoperative LVEF (%)	Preoperative MR	Postoperative complication	ECMO support duration (days)	Postoperative mortality (days)
1	9	40	Mild	Junctional ectopic tachycardia	No	2
2	6	25	Moderate	Ventricular arrhythmia	No	13
3	5	10	Severe	Low cardiac output	23	23
4	18	15	Moderate	Low cardiac output	27	30

LVEF: left ventricular ejection fraction; MR: mitral regurgitation; ECMO: extracorporeal membrane oxygenation

To identify potential predictors of early mortality, key preoperative and operative characteristics were statistically compared between the 4 deceased patients (early mortality group, n = 4) and the survivors (survivor group, n = 19). No significant differences were found between the groups regarding age at surgery, body weight, sex, preoperative MR severity, or the preoperative need for intensive care, inotropic support, or mechanical ventilation (p >0.05). Similarly, cardiopulmonary bypass time and aortic cross-clamp time were comparable between the groups (p >0.05). However, the preoperative LVEF was significantly lower in the early mortality group (median: 10.5, IQR: 26) compared to the survivor group (median: 51.0, IQR: 28) (p = 0.028).

Ventricular function was assessed in 19 patients discharged after surgery. A significant increase in LVEF was observed at 1 month compared to preoperative values (p <0.01). Similarly, LVEF at 6 months was significantly higher than both preoperative and 1-month values (p <0.01). By 6 months postoperatively, all patients had normalized ventricular function. When comparing age groups, infants had lower LVEF values before surgery and at 1 month postoperatively. However, there was no significant difference in LVEF between the groups at 6 months (**Table 3**).

**Table 3:** Ventricular function and severity of MR in surviving patients preoperatively and postoperatively

Clinical parameter	Preoperative	Postoperative (first month)	Postoperative (sixth month)
LVEF total	51 (IQR: 28)	55 (IQR: 22)^a^	65 (IQR: 7)^b^
LVEF infant	40 (IQR: 28)^*^	50 (IQR: 23)^a,*^	65 (IQR: 7)^b^
LVEF older	57 (IQR: 12.5)	60 (IQR: 11.5)^a^	64 (IQR: 8)^b^
MR none, n (%)	2 (10.5%)	2 (10.5%)	5 (26.3%)
MR mild, n (%)	8 (42.1%)	10 (52.6%)	12 (63.2%)
MR moderate, n (%)	7 (36.8%)	6 (31.6%)	2 (10.5%)
MR severe, n (%)	2 (10.5%)	1 (5.3%)	—

*Infant group had significantly lower values than the older group at the same timepoint (p <0.05).

^a^Significantly different from preoperative (p <0.01).

^b^Significantly different from both preoperative and first month values (p <0.01).

LVEF: left ventricular ejection fraction; MR: mitral regurgitation; IQR: interquartile range

Preoperative MR was identified in 17 (89.5%) of the surviving patients. Among them, 9 patients (47.3%) exhibited moderate to severe MR. At the 6-month postoperative follow-up, moderate MR persisted in only 2 patients (10.5%), with no cases of severe MR observed (**Table 3**). Additionally, none of the patients required mitral valvuloplasty during the follow-up period.

Re-intervention was required in 3 patients during the follow-up period due to complications associated with the Takeuchi procedure (pulmonary stenosis or baffle leak), though the assessment of long-term complication rates was not a primary endpoint of this study.

## Discussion

ALCAPA is a rare congenital anomaly of the coronary arteries and a significant, treatable cause of myocardial ischemia and heart failure in children. During fetal life, high pulmonary vascular resistance maintains antegrade flow in the left coronary artery, while adequate pulmonary arterial oxygenation supports normal myocardial perfusion. With the decline in pulmonary vascular resistance during the neonatal period, both pulmonary arterial pressure and antegrade flow in the left coronary artery decrease. As pulmonary arterial oxygen content also diminishes, perfusion of the left ventricular myocardium becomes dependent on collateral vessels arising from the right coronary artery. Patients lacking sufficient collateral supply to the left coronary system often develop early-onset myocardial ischemia, leading to signs of heart failure during infancy. In contrast, patients with adequate collateralization may experience delayed symptom onset or remain clinically silent for years.^5)^ Therefore, patients are mainly classified into 2 groups based on symptom onset and clinical features: infant and adult types. In our study, 12 patients (52.8%) were diagnosed before 1 year of age. These infants commonly presented with respiratory distress and feeding difficulties due to early heart failure. In patients older than 1 year, cardiac murmur and growth delay were the most frequent findings.

ALCAPA is primarily an isolated congenital heart defect. Although rare, it may be associated with simple congenital heart diseases such as atrial septal defect, patent ductus arteriosus, ventricular septal defect, and coarctation of the aorta, as well as complex congenital heart anomalies including Tetralogy of Fallot, Scimitar syndrome, tricuspid atresia, and hypoplastic left heart syndrome.^10,11)^ In our patients, simple congenital heart defects (atrial septal defect and patent ductus arteriosus) were identified. These concomitant defects did not significantly affect the patients’ clinical presentations or prognoses. Complex congenital heart diseases were not detected in any of the cases.

Surgical intervention is recommended for all patients diagnosed with ALCAPA regardless of age or symptoms due to the lifelong risks of ischemia, ventricular arrhythmias, and sudden cardiac death.^12)^ The primary surgical techniques aimed at restoring physiological dual coronary circulation include reimplantation surgery, the Takeuchi procedure, and CABG.^8,13,14)^ In our study, the majority of patients (82.6%) underwent the Takeuchi procedure. This preference may reflect the greater experience of our surgical team with this technique. Although the literature reports a higher rate of reoperation in long-term follow-up after the Takeuchi procedure, it remains a well-established and effective surgical option for this patient population.^15,16)^

The severity of myocardial ischemia is primarily determined by the adequacy and functional capacity of the coronary collateral circulation. In patients with reduced collateral circulation and consequently lower myocardial perfusion, ventricular function is affected at an earlier stage due to ischemia. Clinical signs of heart failure tend to appear sooner, leading to an earlier diagnosis. As a result, patients diagnosed at a younger age exhibit lower LVEF values.^17)^ Consistent with this, our study found that preoperative LVEF was significantly lower in the infant group. We acknowledge that the older patient group contained 2 adult patients (18 and 34 years old), whose adult-type ALCAPA pathology—characterized by superior collateral circulation—typically results in better-preserved preoperative left ventricular function. Their inclusion likely influenced the statistically significant difference in preoperative LVEF observed between the infant and older patient groups and contributed to the overall favorable early recovery of the latter. Due to the rarity of ALCAPA, we maintained this 2-group stratification to preserve statistical power for the core comparison.

Supporting our findings, Cao et al.^18)^ reported no statistically significant differences in cardiopulmonary bypass time and aortic cross-clamp time between patients under 1 year of age and older patients. However, they observed that infants had a significantly longer duration of mechanical ventilation, prolonged stay in the CICU, and extended total hospital stay. In our study, similar results were observed; however, no significant difference in hospital stay duration was found between the 2 patient groups.

In patients undergoing surgical repair for ALCAPA, early mortality rates have been reported as 2.4%–18%, while late mortality is considered rare.^16,19,20)^ Consistent with the literature, the early mortality rate in our patients was 17.4%, whereas no late mortality was observed during follow-up. Younger age at the time of surgery, the severity of left ventricular dysfunction, postoperative moderate or severe MR, and prolonged aortic cross-clamp time have been identified as factors associated with early mortality.^21–23)^ Our comparative analysis found that preoperative LVEF was the only statistically significant predictor of early mortality in our cohort. This finding underscores that the severity of left ventricular dysfunction is the dominant factor determining early outcome. Given the small sample size inherent to this rare condition, our study had low statistical power to detect significance for other established risk factors.

Following the restoration of physiological coronary circulation, myocardial perfusion improves, leading to recovery of ventricular function across all age groups.^24–26)^ Previous studies have reported early improvements in ventricular function, with most patients achieving normalization within 1 year.^27,28)^ In a study by Zhang et al.,^29)^ the median time to normalization of left ventricular function was reported as 6 months. Similarly, in our study, all survivors achieved normal ventricular function by the sixth postoperative month. However, our reliance on M-mode LVEF to assess global function meant that the persistence of subtle regional wall motion abnormalities, particularly in older patients, could not be entirely excluded. This limitation stems from the retrospective design of our study and the use of M-mode LVEF as the sole standard clinical follow-up metric at our institution during the study period. In the infant group, preoperative LVEF values were lower, and due to incomplete recovery, LVEF remained reduced at the first-month postoperative evaluation.

MR in ALCAPA patients typically results from annular dilatation due to left ventricular enlargement and ischemic dysfunction of the papillary muscles. In previous studies, the severity of MR has been reported to decrease postoperatively in most patients, and mitral valve surgery is generally not required except in selected cases.^30,31)^ In a study by Biçer et al.,^32)^ at least moderate MR persisted in patients who initially presented with moderate or greater degrees of regurgitation. Similarly, Sasikumar et al.^33)^ reported that while MR tends to improve in infants, it may worsen or persist in older patients. In our study, although improvement was observed in cases of moderate and severe MR, more than half of the patients still had at least mild to moderate regurgitation during follow-up. However, by the sixth postoperative month, no patient had severe MR, and none required mitral valvuloplasty.

While our small cohort precluded a statistical comparison of functional recovery across surgical techniques, the overarching principle in ALCAPA repair is the successful restoration of a dual-coronary system. The literature confirms that establishing antegrade flow is the principal determinant for the rapid mid-term recovery of LVEF and MR, irrespective of the specific procedure (Takeuchi, reimplantation, or CABG). We therefore attribute the favorable functional outcomes observed in our study primarily to the success of physiological revascularization.

This study provides valuable insights into the surgical outcomes and mid-term cardiac function of patients with ALCAPA. The main limitations of this study are its retrospective design and single-center nature, which may limit the generalizability of the findings. Additionally, the relatively small sample size reflects the rarity of this congenital anomaly and may reduce the statistical power for subgroup analyses.

## Conclusion

ALCAPA is a rare but surgically treatable cause of myocardial ischemia and heart failure in children. Our findings highlight that surgical restoration of physiological coronary circulation leads to significant improvement in ventricular function and MR, particularly within the first 6 months postoperatively. Infants with more severe preoperative dysfunction may experience slower early recovery. Further multicenter prospective studies are warranted to validate these findings and refine treatment strategies.

## Declarations

### Data availability

Underlying data will be made available from the corresponding author upon reasonable request.
